# Supplementary material for: Multiple invasions, Wolbachia and human-aided transport drive the genetic variability of Aedes albopictus in the Iberian Peninsula
Source: Sci Rep. 2022 Nov 30;12:20682. doi: 10.1038/s41598-022-24963-3 (PMC9712423; doi:10.1038/s41598-022-24963-3)
Supplement: Supplementary file 1 — Supplementary Information. [file 41598_2022_24963_MOESM1_ESM.docx]

**Supplementary Information for:**

**Multiple invasions, *Wolbachia* and human-aided transport drive the genetic variability of *Aedes albopictus* in the Iberian Peninsula**

Federica Lucati, Sarah Delacour, John R.B. Palmer, Jenny Caner, Aitana Oltra, Claudia Paredes-Esquivel, Simone Mariani, Santi Escartin, David Roiz, Francisco Collantes, Mikel Bengoa, Tomàs Montalvo, Juan Antonio Delgado, Roger Eritja, Javier Lucientes, Andreu Albó Timor, Frederic Bartumeus, Marc Ventura

**Supplementary Methods**

**Sample collection**

Adult tiger mosquitoes were collected by citizen scientists who participated in the *Mosquito Alert* programme^1^. Mosquito larvae and eggs were collected from road drains and from standard ovitraps, the latter consisting of dark plastic containers filled with water containing a thin wooden blade as oviposition support. Ovitraps were positioned in shaded sites in petrol stations, cemeteries, private properties and public parks. They were inspected weekly or biweekly, wooden boards replaced and the water replenished. Eggs were hatched in the laboratory of the Centre d’Estudis Avançats de Blanes (CEAB-CSIC, Spain), reared to the fourth-larval instar or adult stage and then identified following Schaffner et al.^2^. To minimize bias from sampling related individuals, when possible, no more than three specimens for each ovitrap or road drain were selected for analyses.

**Amplification and sequencing**

Amplifications were done with a total reaction volume of 25 µL, containing 1× PCR buffer (Silverstar, Eurogentec), 1.5 mm MgCl2, 200 µm of each dNTP, 0.2 µM of each primer, 7 µL of template DNA for ITS2 and 2 µL for COI, 1 U Taq polymerase and UV light-sterilized mQ-H2O. PCR amplification for ITS2 primers involved a denaturing step of 1 min at 94°C, five cycles of 40 s at 94°C, 2 min at 37°C and 60 s at 72°C, followed by 35 cycles of 40 s at 94°C, 40 s at 51°C and 60 s at 72°C and a final elongation step of 6 min at 72°C. For COI Folmer, Black, Hoeh, Lutz and Vrijenhoek^3^ primers, PCR amplification involved a denaturing step of 5 min at 95°C, five cycles of 60 s at 95°C, 90 s at 45°C and 45 s at 72°C, followed by 30 cycles of 45 s at 95°C, 45 s at 50°C and 45 s at 72°C and a final elongation step of 7 min at 72°C, while for COI-degenerated primers amplification conditions consisted of 35 cycles of 1 min at 95°C, 2 min at 37°C and 1 min at 72°C. PCR products were purified and sequenced on a ABI 3730XL capillary sequencer by a third party (Macrogen, Seoul, South Korea).

**Construction of covariates: potential tiger mosquito flux**

The *Ae. albopictus* risk estimates used in the tiger mosquito flux variable were made with a Bayesian multilevel logistic regression of *Ae. albopictus* presence in Spain measured as expert-validated *Mosquito Alert* reports of adult *Ae. albopictus* at given points in time and space from 2014 through 2021. We combined these reports of presence with pseudoabsences created by placing points randomly within the same geographic and temporal bounds. The space-time region within these bounds was divided into sampling-cell-days, using the 0.025 degree latitude by 0.025 degree longitude sampling cells within which the *Mosquito Alert* app collects anonymous background tracks and estimates sampling effort based on the number of citizen scientists in each cell and the amount of time elapsed since each downloaded the app (since participant motivation has been observed to drop over time)^1^. The number of pseudoabsences placed in each sampling-cell-day was proportional to the *Mosquito Alert* sampling effort. The model includes random intercepts for municipalities and province-years to capture spatio-temporal variation in the observed *Ae.* *albopictus* distribution. It includes random intercepts for landcover, constructed from the 2018 Corine Landcover dataset, and it includes a random slope at the province level for the Mosquito Weather Index, developed by the LIFE CONOPS project^4^ and constructed using the mean monthly temperature, humidity, and wind velocity variables from the ERA5 dataset^5^. The model also includes an offset for sampling effort and random intercepts at the sampling cell level to control for variation and anomalies in sampling behaviour by the citizen scientists beyond what is captured by the placement of pseudoabsences.

The *Ae. albopictus* risk estimates were combined with the estimated daily number of people commuting between each pair of municipalities in either direction (we use commuting in either direction because mosquitoes can potentially be transported from one municipality to another on the way to work or on the way home, and because the flux measure of interest for genetic distance is the total bidirectional flux). These commuting estimates were made using data from the LFS, a continuous survey of the Spanish population living in family dwellings, which is administered four times per year using a two-stage stratified random sample with approximately 160,000 respondents per wave^6^. The survey includes questions about each respondent’s home province and the province in which they work, which can be used to estimate commuter fluxes between provinces^7,8^. Inter-province commuter fluxes were estimated on a quarterly basis directly from the LFS micro-data using the survey weights to make population level estimates, and calculating mean fluxes per year. These estimates were then down-scaled to the municipality level using administrative data on places of domicile. Inter-province fluxes were distributed across municipalities in proportion to the relative residential populations in each municipality, based on the assumption that inter-province commuters’ homes are distributed across municipalities in their home province similarly to the rest of the population’s homes in that province, and that inter-province commuters’ places of work are distributed across municipalities in their work province similarly to the rest of the population’s homes in that province^7^. The inter-municipality fluxes are shown in Supplementary Fig. S4.

**Model fitting and comparison**

Models were fit using Hamiltonian Monte Carlo Markov (HMC) sampling implemented by Stan^9^ using the brms package for R^10^. Computation was done on the CEAB-CSIC’s high-performance computational cluster, using 48 cores and within-chain parallelization implemented by the cmdstanr package for R^11^. All independent variables were centered and standardized before model fitting. We compared models using leave-one-out cross validation (LOO)^12,13^ and Bayesian R-squared^14^. Given the large size of the dataset, we carried out LOO using subsamples of the data in order to speed computation, as described in Magnusson, Andersen, Jonasson and Vehtari^12^. LOO was then estimated with the Pareto-smoothed importance sampling proposed by Vehtari, Gelman and Gabry^13^. Expected log pointwise predictive density (ELPD) was used as a measure of predictive accuracy. This analysis was implemented using the loo^13,15^ and brms packages in R. As for the R-squared, we used the Bayesian version of R-squared proposed by Gelman, Goodrich, Gabry and Vehtari^14^ to avoid the problem that the numerator may be larger than the denominator in Bayesian models. We implemented this using the brms package in R.

**Supplementary figures**


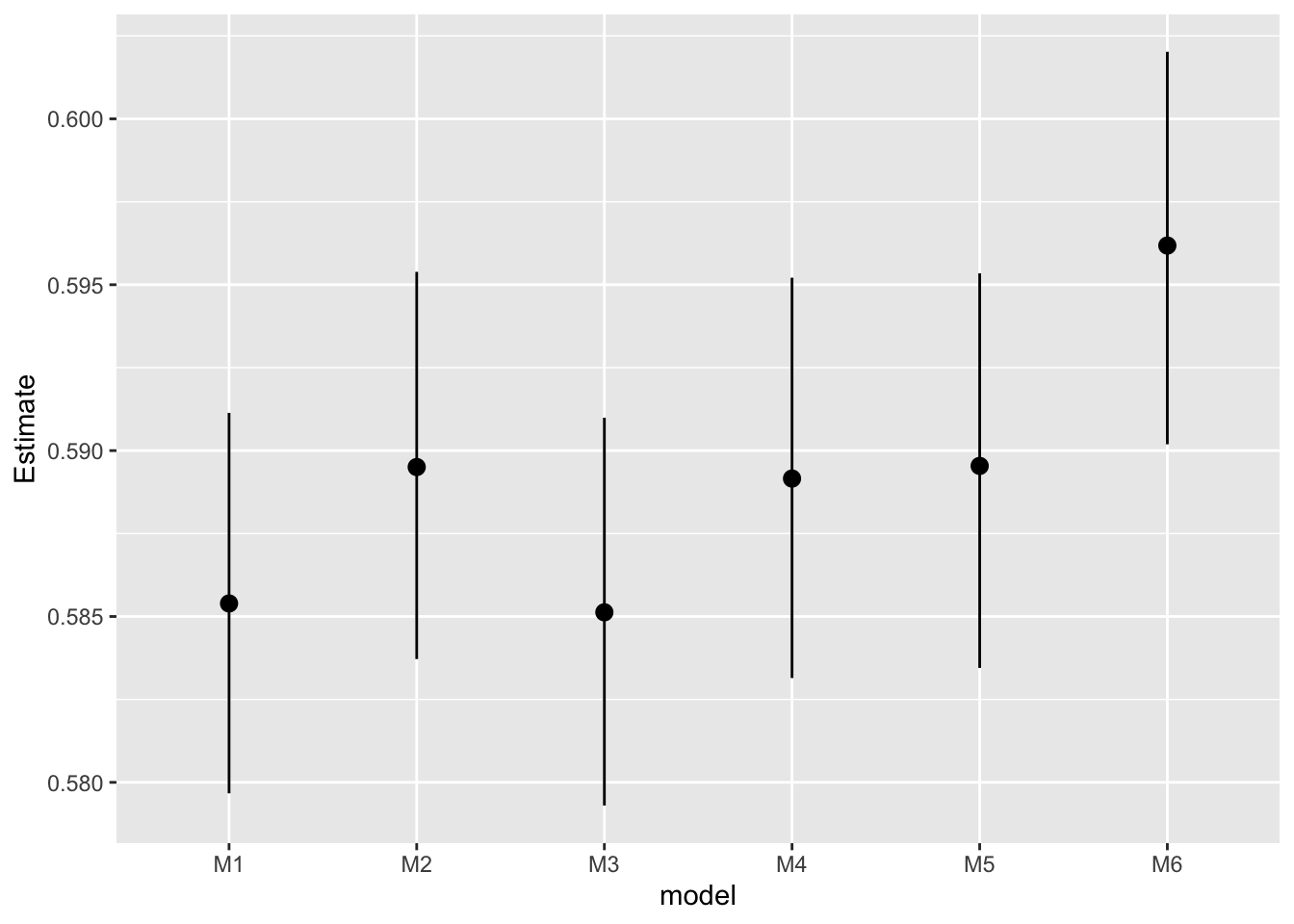


**Supplementary Figure S1.** Comparison of zero-inflated beta regression models based on Bayesian R-squared. Points indicate the R-squared estimate for each model (M1-M6). Lines indicate the 95% credible interval.


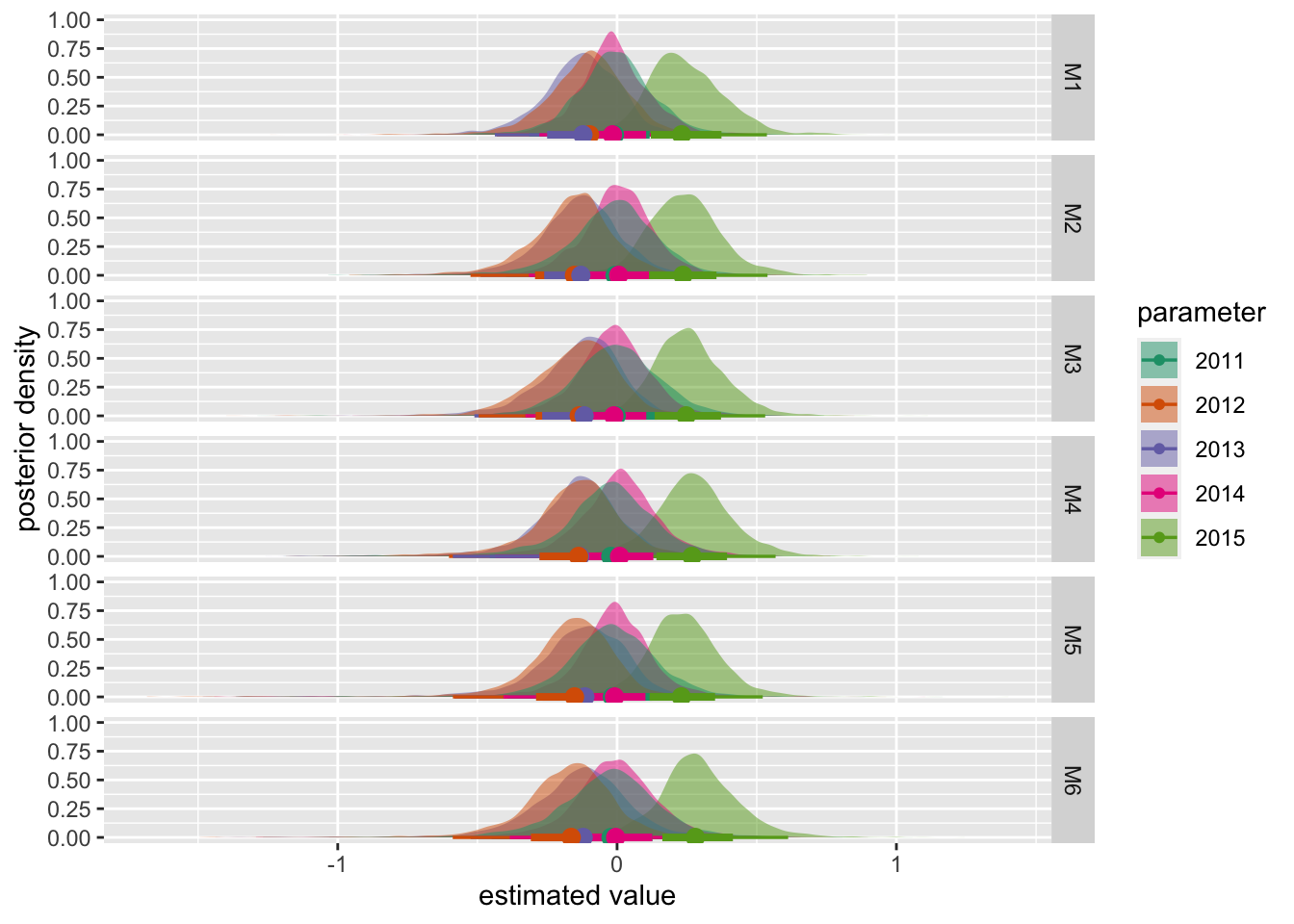


**Supplementary Figure S2.** Posterior distributions for year random intercepts in the zero-inflated beta regression models (M1-M6) of the beta mean (*μ*).


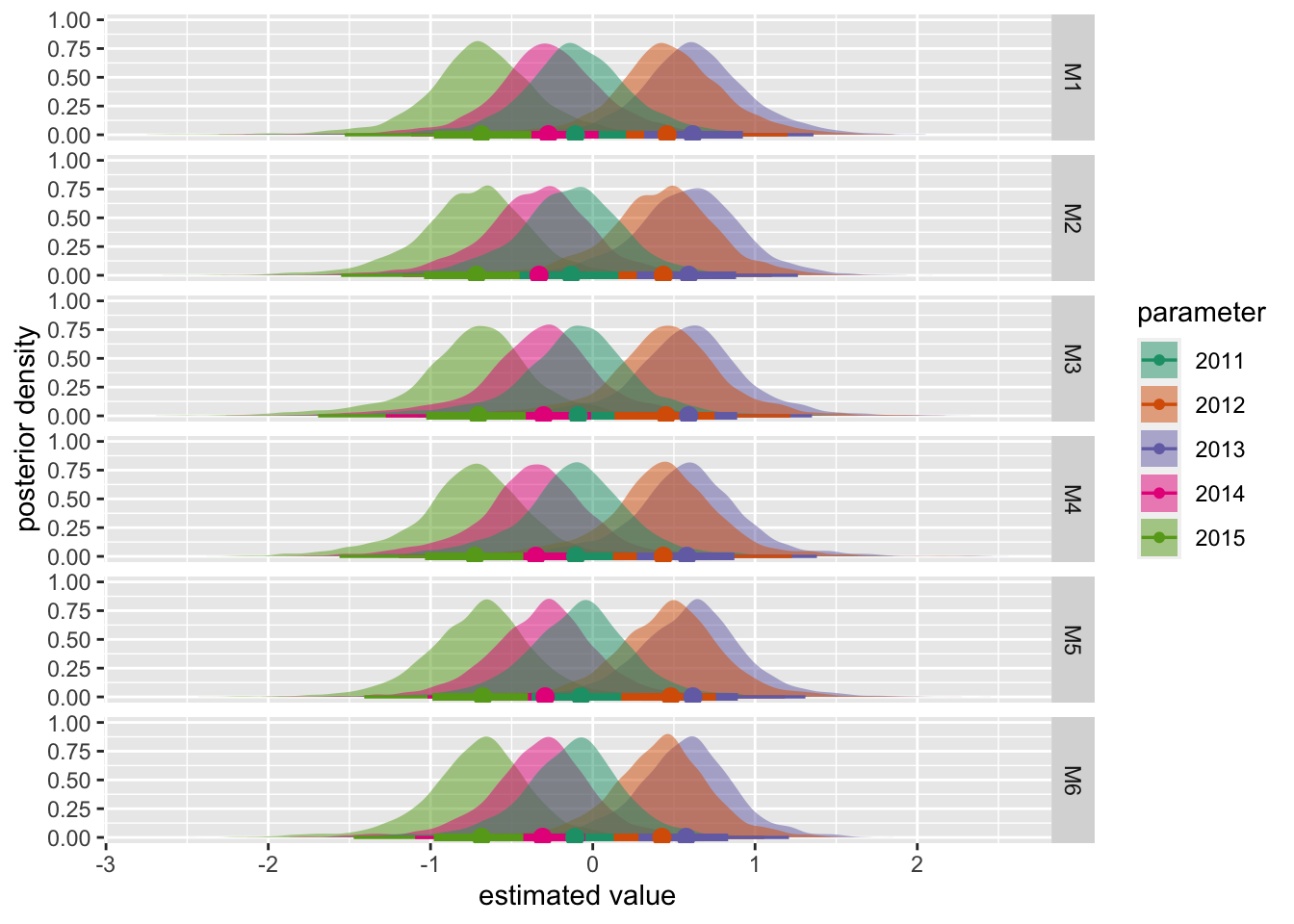


**Supplementary Figure S3.** Posterior distributions for year random intercepts in the zero-inflated beta regression models (M1-M6) of the zeros.


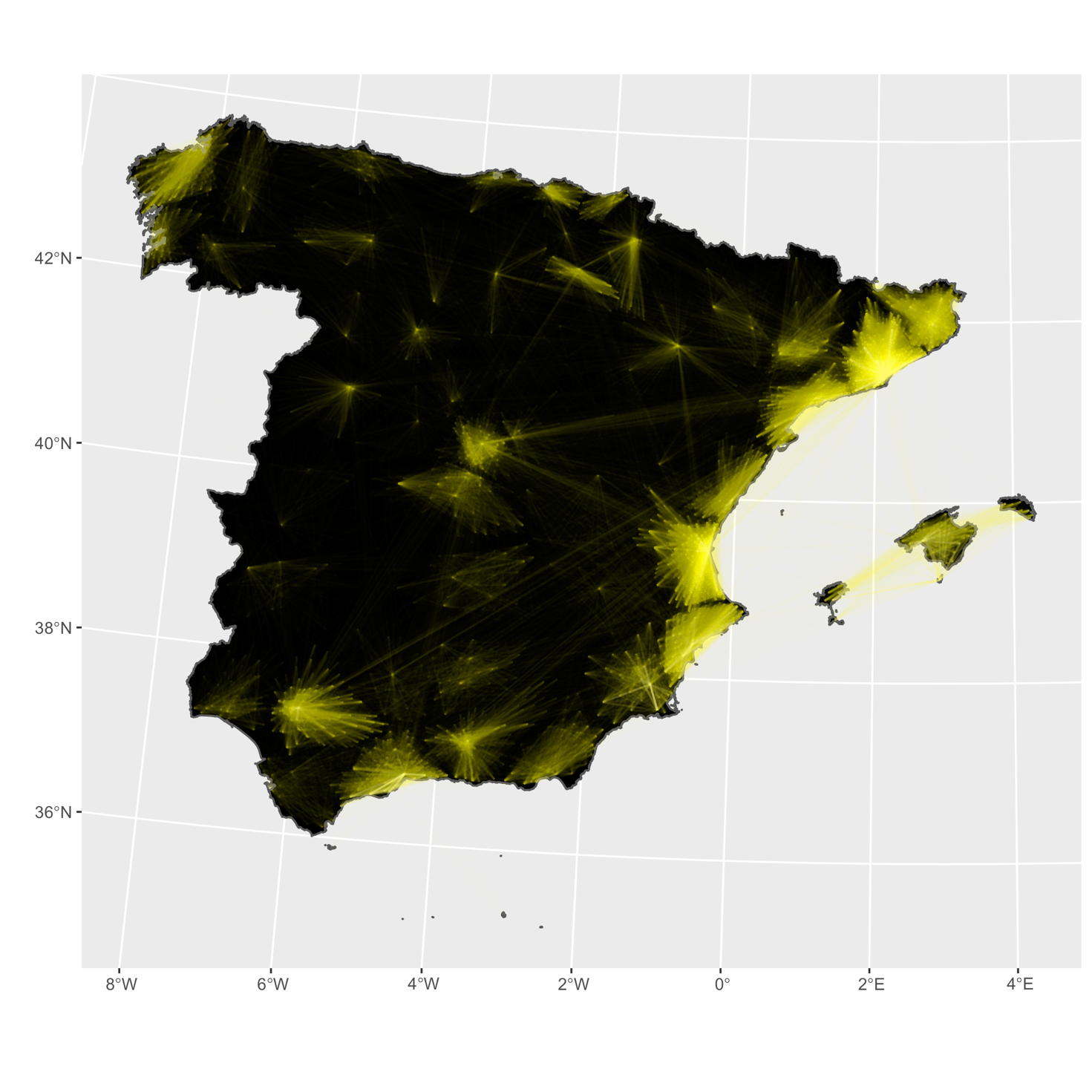


**Supplementary Figure S4.** Map of potential tiger mosquito flux between municipalities in Spain. Brighter yellow represents higher flux. Lines drawn only between municipality pairs with estimated flux of at least two tiger mosquitoes per day.

**Supplementary tables**

**Supplementary Table S1.** Comparison of zero-inflated beta regression models based on expected log pointwise predictive density (ELPD) using leave-one-out cross-validation (LOO). Abbreviations: elpd_diff – ELPD point difference relative to Model 6 (M6); se_diff – Standard Error point difference relative to M6.

|  | elpd_diff | se_diff |
| --- | --- | --- |
| M6 | 0 | 0 |
| M1 | 138 | 20 |
| M5 | 166 | 16 |
| M3 | 219 | 20 |
| M2 | 271 | 24 |
| M4 | 283 | 23 |

**Supplementary Table S2.** Parameter estimates for the six zero-inflated beta regression models (M1-M6) and comparison of models based on Bayesian R-squared and expected log pointwise predictive density (ELPD) using leave-one-out cross-validation (LOO). Abbreviations: Int. - random intercepts; sp_prox - spatial proximity; ln_mosq_flux – natural log of potential tiger mosquito flux plus 1; yr_diff – temporal distance; sp_dist - spatial distance; SE - Standard Error.

|  | **M1** | **M2** | **M3** | **M4** | **M5** | **M6** |
| --- | --- | --- | --- | --- | --- | --- |
| Int. | -5.13 (0.14) | -5.12 (0.14) | -5.14 (0.14) | -5.13 (0.17) | -5.11 (0.18) | -5.12 (0.18) |
| sp_prox | -0.02 (0.00) |  | -0.02 (0.00) |  | -0.01 (0.00) | -0.03 (0.00) |
| ln_mosq_flux |  | -0.02 (0.00) |  | -0.01 (0.00) | -0.02 (0.00) | -0.02 (0.00) |
| yr_diff |  |  | 0.01 (0.00) | 0.01 (0.00) | 0.01 (0.00) | 0.01 (0.00) |
| sp_dist |  |  |  |  |  | -0.02 (0.00) |
| Bayes. R-sq. | 0.5854 | 0.5895 | 0.5851 | 0.5892 | 0.5895 | 0.5962 |
| SE Bayes. R-sq. | 0.0029 | 0.0030 | 0.0030 | 0.0031 | 0.0030 | 0.0030 |
| ELPD | 206033.22 | 205899.82 | 205952.06 | 205887.55 | 206004.83 | 206170.78 |
| SE ELPD | 890.93 | 894.88 | 895.98 | 895.40 | 894.05 | 892.85 |
| N | 87153.00 | 87153.00 | 87153.00 | 87153.00 | 87153.00 | 87153.00 |

**References**

1 Palmer, J. R. *et al.* Citizen science provides a reliable and scalable tool to track disease-carrying mosquitoes. *Nat. Commun.* **8**, 1-13 (2017).

2 Schaffner, F. *et al.* *Les moustiques d'Europe: logiciel d'identification et d'enseignement = The mosquitoes of Europe: an identification and training programme*. (IRD Editions & EID Méditerranée, 2001).

3 Folmer, O., Black, M., Hoeh, W., Lutz, R. & Vrijenhoek, R. DNA primers for amplification of mitochondrial cytochrome *c* oxidase subunit I from diverse metazoan invertebrates. *Mol. Mar. Biol. Biotechnol.* **3**, 294–299 (1994).

4 Badieritakis, Ε. *et al.* *Aedes albopictus* (Skuse, 1895)(Diptera: Culicidae) in Greece: 13 years of living with the Asian tiger mosquito. *Parasitol. Res.* **117**, 453-460 (2018).

5 Hersbach, H. *et al.* The ERA5 global reanalysis. *Q. J. R. Meteorol. Soc.* **146**, 1999-2049 (2020).

6 Instituto Nacional de Estadistica (INE). Labor Force Survey: Sample Design and Evaluation of Data Quality. (Department of Survey Sample Design, Madrid, 2016).

7 Eritja, R., Palmer, J. R., Roiz, D., Sanpera-Calbet, I. & Bartumeus, F. Direct evidence of adult *Aedes albopictus* dispersal by car. *Sci. Rep.* **7**, 1-15 (2017).

8 Tizzoni, M. *et al.* On the use of human mobility proxies for modeling epidemics. *PLoS Comp. Biol.* **10**, e1003716 (2014).

9 Stan Development Team. *Stan Modeling Language Users Guide and Reference Manual. Version 2.29.*, <<https://mc-stan.org>> (2022).

10 Bürkner, P.-C. brms: An R package for Bayesian multilevel models using Stan. *J. Stat. Softw.* **80**, 1-28 (2017).

11 Gabry, J. & Češnovar, R. *cmdstanr: R Interface to’CmdStan’*, <<https://mc-stan.org/cmdstanr>> (2020).

12 Magnusson, M., Andersen, M., Jonasson, J. & Vehtari, A. Bayesian leave-one-out cross-validation for large data. in *International Conference on Machine Learning.* 4244-4253 (PMLR).

13 Vehtari, A., Gelman, A. & Gabry, J. Practical Bayesian model evaluation using leave-one-out cross-validation and WAIC. *Stat. Comput.* **27**, 1413-1432 (2017).

14 Gelman, A., Goodrich, B., Gabry, J. & Vehtari, A. R-squared for Bayesian regression models. *Am. Stat.*, doi:10.1080/00031305.2018.1549100 (2019).

15 Yao, Y., Vehtari, A., Simpson, D. & Gelman, A. Using stacking to average Bayesian predictive distributions (with discussion). *Bayesian Anal.* **13**, 917-1007 (2018).
